# Supplementary material for: Synthetic Pseudo-Spin-Hall effect in acoustic metamaterials
Source: Nat Commun. 2022 Oct 25;13:6332. doi: 10.1038/s41467-022-34072-4 (PMC9596417; doi:10.1038/s41467-022-34072-4)
Supplement: Supplementary file 1 — Supplementary materials [file 41467_2022_34072_MOESM1_ESM.pdf]

Supplementary Information

Synthetic Pseudo-Spin-Hall Effect in Acoustic Metamaterials

Matthew Weiner[1,2,3], Xiang Ni[1,2,4], Andrea Alù[4,1,2], and Alexander B. Khanikaev[1,2,3]

[1]Department of Electrical Engineering, Grove School of Engineering, City College of the City University of New York, 140th Street and Convent Avenue, New York, NY 10031, USA.

[2]Physics Program, Graduate Center of the City University of New York, New York, NY 10016, USA.

[3]Department of Physics, City College of New York, 160 Convent Ave., New York, NY 10031, USA

[4]Advanced Science Research Center, City University of New York, New York, NY 10031, USA

Section 1 – Derivation of dispersion relation for guided modes

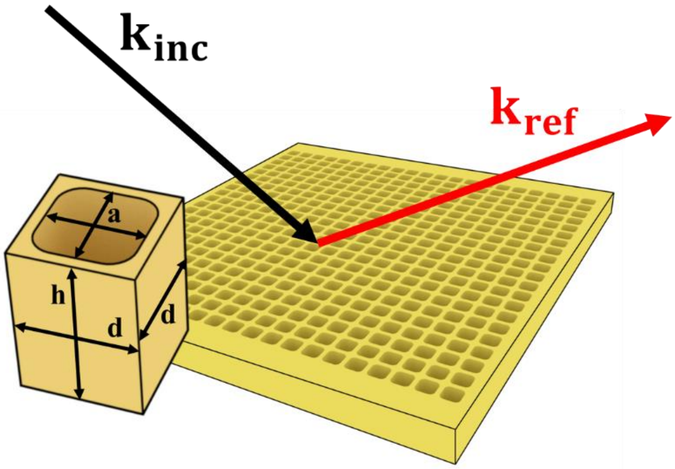

Figure S1 – 2D array of holes

Consider a two-dimensional hole array shown in figure S1 with an incoming acoustic pressure plane wave. We consider two regions: the fields above the holes (region I) and the fields inside the holes (region II). The solutions for the pressure fields in the two regions are given as

$$p_I = p_{inc} e^{i\left(k_x^{(0)} x + k_y^{(0)} y + k_z^{(0)} z\right)} + \sum_{mn} p_{mn} e^{i\left(k_x^{(m)} x + k_y^{(n)} y - k_z^{(mn)} z\right)}$$

$$p_{II} = \left[C^+ e^{i\,k_0 z} + C^- e^{-i\,k_0 z}\right][\theta(x) - \theta(x-a)][\theta(y) - \theta(y-a)]$$

Here, $C^{\pm}$ are undetermined coefficients, θ is the Heaviside step function, and p_n are the harmonic expansion coefficients of the reflected wave. The pressure waves are subject to the following boundary conditions:

$$p_I(z=0) = p_{II}(z=0) \qquad \text{(Continuity of the pressure field)}$$

$$\hat{z} \cdot \nabla p_I(z=0) = \hat{z} \cdot \nabla p_{II}(z=0) \qquad \text{(Continuity of the velocity field)}$$

$$\hat{z} \cdot \nabla p_{II}(z=h) \ = 0 \qquad \text{(Hard wall boundary condition)}$$

After applying the boundary conditions, we find the following expression for p_n:

$$p_n = -\left[\frac{k_0}{k_z^{(mn)}} \frac{2i\ \mathrm{Tan}(k_0 h) S_{00} S_{mn}}{1 - i\ \mathrm{Tan}(k_0 h) \sum_{m'n'} \frac{k_0}{k_z^{(m'n')}} S_{m'n'}^2} + \delta_{mn,00}\right] p_{inc}$$

Where

$$S_{mn} = \frac{1}{ad} \int_{-a/2}^{a/2} \int_{-a/2}^{a/2} dx\, dy\, e^{-ik_x^{(m)} x - ik_y^{(n)} y}$$

We can reasonably approximate this expression by claiming that the zeroth order is the dominant mode of the reflected wave and simplify:

$$\frac{p_0}{p_{inc}} = -\frac{1 + i \frac{k_0}{k_z} S_0^2\ \mathrm{Tan}(k_0 h)}{1 - i \frac{k_0}{k_z} S_0^2\ \mathrm{Tan}(k_0 h)}$$

For the guided mode, we require that $k_x^2 + k_y^2 > k_0^2$ such that the radicand of the expression $k_z = \sqrt{k_0^2 - k_x^2 - k_y^2}$ is explicitly negative, so $k_z = i\sqrt{k_x^2 + k_y^2 - k_0^2}$. This places the modal dispersion below the sound line, exactly as required for a guided mode. And finally, to solve for the dispersion, we simply find where the above expression diverges for $k_x > k_0$, so setting the denominator equal to zero yields:

$$\frac{\sqrt{k_x^2 + k_y^2 - k_0^2}}{k_0} = S_0^2\ \mathrm{Tan}(k_0 h)$$

With the consideration that $k_0 = \frac{\omega}{c}$, we obtain the dispersion relation as shown in Fig. 1B. Interestingly, it should be noted that this is the exact dispersion relation obtained for the electromagnetic analogue.

Section 2 – Distance above the hole of circular polarization hot spot as a function of k

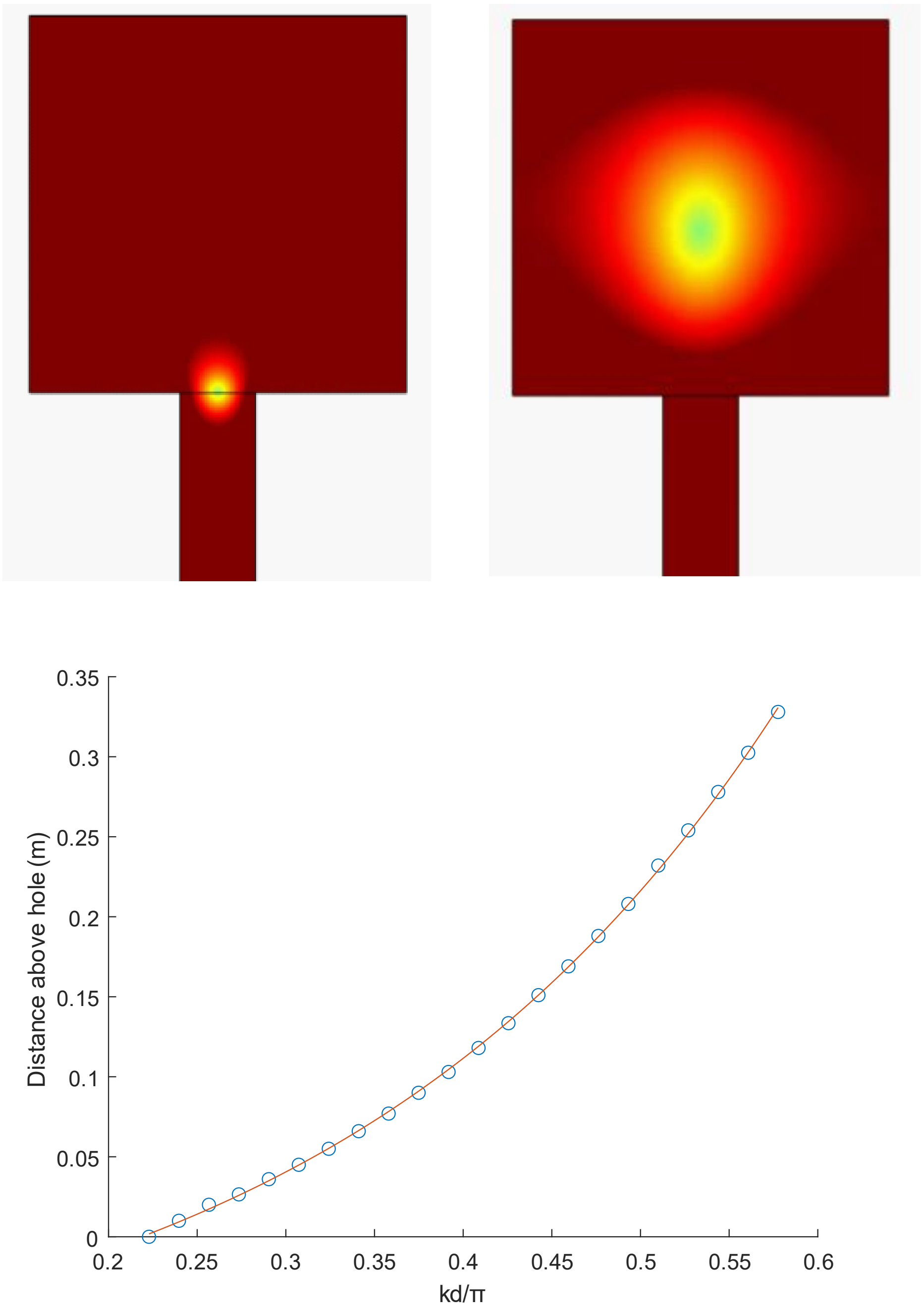

Figure S2 – Top left: circular polarization hot spot at $\frac{kd}{\pi} = 0.23$. Before this value, the hot spot is inside of the hole. As you increase the k-value, the hot spot rises above the hole (top right). Bottom: Distance above the hole as a function of the k-value. As you approach a wavenumber that is parallel to the surface, the hot spot extends exponentially upward towards infinitely far away.

Section 3 - 45° ($\Gamma - \mathrm{M}$) high symmetry directional excitation

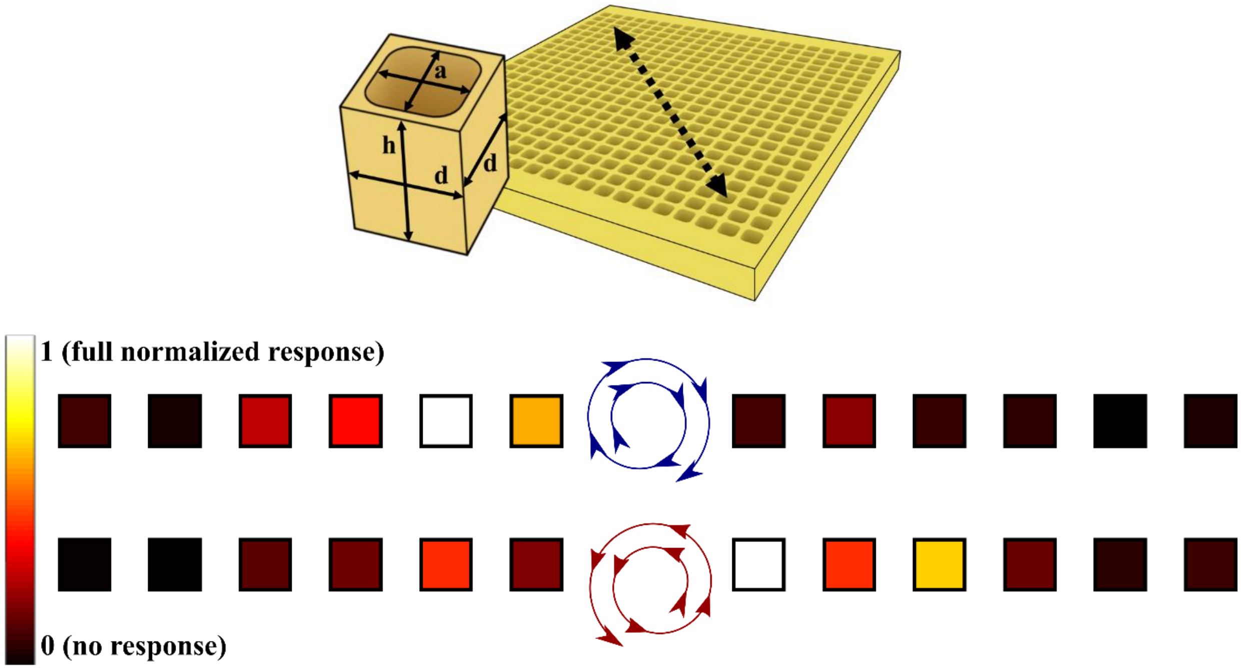

Figure S3 – Top: Dashed line showing the directional propagation along the 45° direction. Bottom: Experimental results along the dashed line demonstrating directional excitation.
